# A genome‐wide analysis of long noncoding RNA profile identifies differentially expressed lncRNAs associated with Esophageal cancer

**DOI:** 10.1002/cam4.1536

**Published:** 2018-06-21

**Authors:** Wenjia Liu, Yiyang Zhang, Min Chen, Liangliang Shi, Lei Xu, Xiaoping Zou

**Affiliations:** ^1^ Department of Gastroenterology Nanjing Drum Tower Hospital Clinical College of Nanjing Medical University Nanjing China; ^2^ Department of Gastroenterology Nanjing Drum Tower Hospital The Affiliated Hospital of Nanjing University Medical School Nanjing China

**Keywords:** cell proliferation, DUXAP8, esophageal cancer, lncRNAs, profiling

## Abstract

Esophageal cancer is one of the most common cancers and a leading cause of cancer‐related death worldwide. However, the mechanism of esophageal cancer pathogenesis remains poorly understood. Long noncoding RNAs (lncRNAs) dysregulation have been reported to involve in various human cancers, which highlights the potential of lncRNAs used as novel biomarkers for cancer diagnosis. Although more efforts have been made to identify novel lncRNAs signature in esophageal cancer, the expression pattern, prognostic value, and biological function of most lncRNAs in esophageal cancer still need to be systematically investigated. In this study, we comprehensively analyzed the expression profile of lncRNAs in more than 200 esophageal cancer patients tissue samples from The Cancer Genome Atlas (TCGA) and Gene Expression Omnibus (GEO). We identified thousands of lncRNAs are differentially expressed in esophageal cancer tissues, and many of those lncRNAs expression are associated with patients overall survival or recurrence‐free survival time. Moreover, copy number variation analyses revealed that genomic loci copy number amplification or deletion might contribute to these lncRNAs dysregulation. Among these lncRNAs, DUXAP8 and LINC00460 were significantly upregulated, and GO enrichment analyses indicated that the two lncRNAs associated protein‐coding genes involve with many known biological processes, such as cell cycle and cell‐cell adherens junction. Further experimental validation revealed that knockdown of DUXAP8 could impair esophageal cancer cells proliferation and invasion in vitro. Taken together, our findings identified more aberrantly expressed lncRNAs in esophageal cancer that may provide a useful resource for identifying novel esophageal cancer associated lncRNAs.

## 1 INTRODUCTION

Esophageal cancer is one of the most common malignant tumors and the leading cause of cancer‐related death worldwide.^1^, ^2^ The two major subtypes of esophageal cancer are esophageal squamous cell carcinoma (ESCC) and Esophageal adenocarcinoma (EAD).^3^ In spite of the advance on molecular diagnostic techniques, surgical treatment, chemotherapy, molecular targeted therapy, the 5 years overall survival rate remains <25% because most of the esophageal cancer patients are diagnosed at a late stage with a poor prognosis.^4^, ^5^ Lacking of specific symptoms and effective therapeutic targets has been the main obstacles of developing novel therapeutic programs and improving patient prognosis and survival time. Therefore, a better understanding the molecular mechanisms of esophageal cancer tumorigenesis and identifying new biomarkers are critical for the improvement of early diagnosis and therapy of esophageal cancer.

The occurrence of cancer is caused by variety of factors, and genetic changes are regarded to involve in this pathological process. Recently, a growing number of studies reveal that noncoding RNAs (ncRNAs) as well as protein‐coding genes play important roles in human diseases, especially cancers.^6^, ^7^, ^8^ long noncoding RNAs (lncRNAs) are the new member of ncRNA with a length >200 nt, and lacking of protein‐coding capacity.^9^ To date, increasing evidence have demonstrated that lncRNAs are widely expressed in most of organ tissues, and participate in various cellular biological process, including X chromatin imprinting, muscle cells differentiation, immune response, cell fate decision, tumor cell proliferation, and drug resistance.^10^, ^11^ Importantly, RNA sequencing and microarray analyses of tumor and normal tissues in diverse types of human cancers revealed that thousands of lncRNAs are dysregulated in cancer tissues, and part of these lncRNAs are emerging as key molecules during carcinogenesis.^12^, ^13^ lncRNAs involve in the tumorigenesis by functioning as oncogenes or tumor suppressors through chromatin remodeling, histone modification, acting as sponge for microRNAs, and affecting RNA stability.^14^, ^15^ For example, Sun and colleagues report that gastric cancer associated lncRNA HOXA11‐AS promotes cell growth and metastasis through functioning as scaffold of EZH2 and LSD1 and acting as competing endogenous RNA (ceRNA) for miR‐1297.^16^

In case of esophageal cancer, the involvement of several lncRNAs in esophageal cancer and their underlying mechanisms have been reported.^17^, ^18^ For instance, the lncRNA cancer susceptibility candidate 9(CASC9) is significantly upregulated in ESCC tissues, and over‐expressed CASC9 promotes ESCC cells growth through interacting with EZH2 and repressing PDCD4 transcription.^19^ In addition, HOTAIR promotes cell invasion and metastasis, and epithelial to mesenchymal transition by acting as a miR‐148a sponge to regulate Snail2 expression in esophageal cancer.^20^ Moreover, over‐expressed HOTTIP promotes cell proliferation and metastasis through acting as a miR‐30b sponge and interacting with WDR5 to modulate the expression of HOXA13 and Snail 1in ESCC cells.^21^ In addition, LUCAT1 was found to be significantly upregulated in ESCC, and promote the formation and metastasis of ESCC through regulating the stability of DNMT1 and repressing the expression of tumor suppressors.^22^ Although a few individual lncRNAs were characterized, most of the lncRNAs expression pattern and their biological function in esophageal cancer remains unclear. To determine the lncRNAs profiling and identify esophageal cancer associated lncRNAs, we analyzed differentially expressed lncRNAs in human esophageal cancer samples and adjacent nontumor samples by performing genome‐wide comprehensively analyses using TCGA RNA sequencing data and public microarray data from GEO. This study reveals the differentially expressed lncRNAs in esophageal cancer, which provides a useful list of lncRNAs candidates for esophageal cancer diagnosis and therapy.

## 2 METHODS AND MATERIALS

### 2.1 TCGA and public microarray data analysis

The TCGA esophageal cancer tissue and adjacent nontumor tissue samples RNA sequencing data and corresponding clinical information were obtained from https://gdac.broadinstitute.org/. Four public esophageal cancer microarray profiling datasets (GSE45670,^23^ GSE53622,^12^ GSE89102, GSE92396) were downloaded from the Gene Expression Omnibus (GEO). lncRNAs profiling of GEO microarray datasets was annotated and analyzed based on the Affymetrix Human Genome U133 Plus 2.0 Array and U133A Array, Agilent‐038314 CBC Homo sapiens lncRNA + mRNA microarray V2.0, Agilent‐045997 Arraystar human lncRNA microarray V3, and Affymetrix Human Gene 1.0 ST Array platforms. All these TCGA RNA sequencing, gene, or lncRNA microarray data were preprocessed using R software and packages.

### 2.2 lncRNAs copy number variation analysis

The raw TCGA esophageal cancer tissues somatic gene copy number variation data were downloaded from Broad GDAC FireBrowser (https://gdac.broadinstitute.org/). Then, all of lncRNAs genomic regions were mapped to the GISTIC peaks, and GISTIC 2.0 was used to annotate and determine the significant recurrent of each lncRNAs genomic loci copy number amplifications or deletions. Next, the peaks of amplification and deletion with *q* values <0.25 were defined as significant. The number of lncRNAs in peaks, focal/broad frequencies, and *q* values of each peak were summarized at gene level.

### 2.3 Esophageal cancer survival associated lncRNAs analyses

To explore the relationship between lncRNAs expression levels and esophageal cancer patients survival, the univariable Cox regression analysis was performed. Next, univariable Cox regression analyses were conducted to determine each of those lncRNAs as dependent variable factor. Then, the esophageal cancer patients were divided into high‐ or low‐lncRNA expression groups based on the median expression level of each lncRNA. Then, the lncRNA with log‐rank *P* value <.05 between high‐ and low‐expression groups were considered statistically significant. All the analyses were conducted using R software and Bio‐conductor.

### 2.4 Cell culture and siRNA transfection

Esophageal cancer cell lines KYSE510 and KYSE30 were purchased from the Type Culture Collection of the Chinese Academy of Sciences (Shanghai, China). KYSE510 and KYSE30 cells were cultured in Roswell Park Memorial Institute (RPMI) 1640 Medium (Invitrogen, Shanghai, China) supplied with 10% fetal bovine serum (Invitrogen, Shanghai, China), 100 U/mL penicillin and streptomycin (Invitrogen), at 37°C with 5% CO_2_. The small interfering RNAs for DUXAP8 and negative control (Invitrogen, Carlsbad, CA) were transfected into KYSE510 and KYSE30 cells using RNAiMAX (Invitrogen) based on the manufacturer's instructions. 48 hours after transfection, the KYSE510 and KYSE30 cells were harvested for RNA extraction. The siRNA sequences of DUXAP8 are 5′‐AAGATAAAGGTGGTTTCCACAAGAA‐3′ and 5′‐ CAGCATACTTCAAATTCACAGCAAA‐3′.

### 2.5 RNA extraction and qRT‐PCR analyses

The total RNA of KYSE510 and KYSE30 cells was extracted using RNeasy Purification Kit (QIAGEN) according to the manufacturer's manual. Then, 1 μg of total RNA was reverse transcribed into cDNA using PrimeScript RT Reagent Kit (TaKaRa, Dalian, China). SYBR Premix Ex Taq (TaKaRa) was used to examine the DUXAP8 expression, and GAPDH was used as internal control. The DUXAP8 primer is forward 5′‐AGGATGGAGTCTCGCTGTATTGC‐3′ and reverse 5′‐ GGAGGTTTGTTTTCTTCTTTTTT‐3′. The GAPDH primer sequence is forward 5′‐ AGAAGGCTGGGGCTCATTTG‐3′ and reverse 5′‐ AGGGGCCATCCACAGTCTTC‐3′. qRT‐PCR analyses were performed on CFX96 Touch Real‐Time PCR Detection System (Bio‐Rad Hercules, CA, USA), and comparative cycle threshold (CT) (2−ΔΔCT) method was used to analyze the qRT‐PCR data.

### 2.6 CCK8 cell proliferation analyses

The KYSE510 and KYSE30 cells were transfected with DUXAP8 or negative control siRNAs. 48 hours after transfection, the KYSE510 and KYSE30 cells were seeded into 96‐well plates with 2000 cells/well. Then, 10 μL CCK8 regent was added into each well. After incubation for 3 hours, the absorbance value of OD450 was obtained. The assays were conducted every 24 hours.

### 2.7 Statistical analysis

The one‐way ANOVA and Students *t* test (2‐tailed) methods were used to analyze the qRT‐PCR and in vitro CCK8 assays data using SPSS 17.0. *P* value <.05 was considered statistically significant.

## 3 RESULTS

### 3.1 Identification of differentially expressed lncRNAs in esophageal cancer tissues

**Figure 1:**
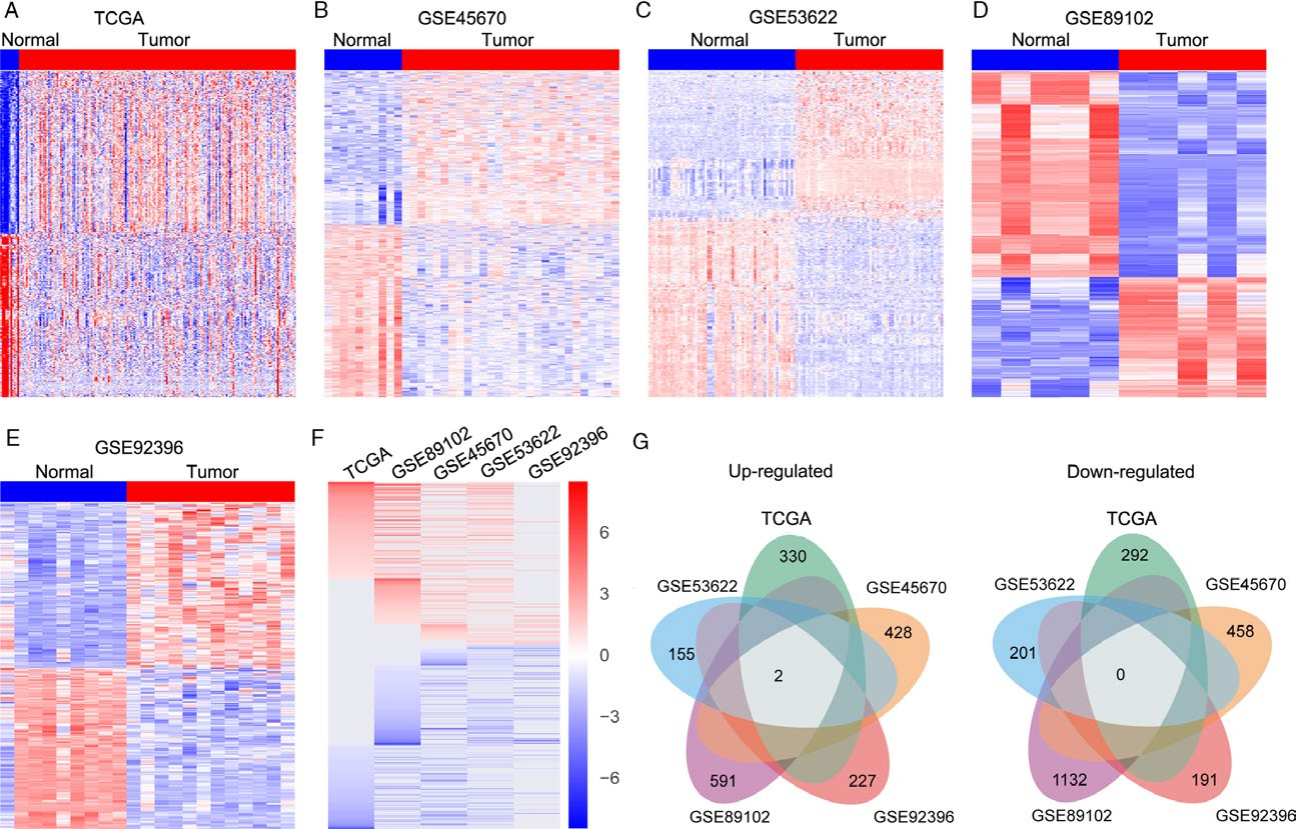
The lncRNAs profiling in human esophageal cancer. A, A heat map was drawn to show the differentially expressed lncRNAs in esophageal cancer tissues and normal tissue samples in the TCGA RNA sequencing data. B‐E, Heat maps were drawn to show the differentially expressed lncRNAs in esophageal cancer tissues and normal tissue samples in the GSE45670, GSE53622, GSE89102, GSE92396 datasets. F, A heat map was drawn to show the differentially expressed lncRNAs (consistently upregulated or downregulated at least two datasets, fold change) in TCGA, GSE45670, GSE53622, GSE89102, and GSE92396 datasets. G, Venn diagram of dysregulated lncRNAs profiling in TCGA, GSE45670, GSE53622, GSE89102, and GSE92396 datasets

To identify the differentially expressed lncRNAs in esophageal cancer tissues, we firstly downloaded the TCGA esophageal cancer and normal tissue samples RNA sequencing data and four microarray datasets (GSE45670, GSE53622, GSE89102, and GSE92396) from GEO. The TCGA data consist of 369 esophageal cancer samples and 24 normal tissue samples, while the GSE45670 dataset consists of 28 tumor and 10 normal samples; GSE53622 consists of 60 paired esophageal cancer samples; GSE89102 consists of five paired esophageal cancer samples; GSE92396 consists of 12 tumor and nine normal samples. Further analyses of these data showed that 4534 lncRNAs were differentially expressed in the TCGA dataset (556 upregulated and 489 downregulated); 1275 lncRNAs were dysregulated in the GSE45670 dataset (602 upregulated and 673 downregulated); 743 lncRNAs were differentially expressed in the GSE53622 dataset (335 upregulated and 408 downregulated); 2214 lncRNAs were differentially expressed in the GSE89102 dataset (817 upregulated and 1397 downregulated); and 564 lncRNAs were dysregulated in the GSE92396 dataset (285 upregulated and 279 downregulated) (Figure 1A‐E, and Table S1). Further Venn analyses revealed that 378 lncRNAs were consistently upregulated and 431 lncRNAs were downregulated in at least two datasets (Figure 1E,F, Table S2). These findings indicate that thousands of lncRNAs are deferentially expressed in esophageal cancer, and part of those lncRNAs may be useful biomarkers for esophageal cancer diagnosis.

**Figure 2:**
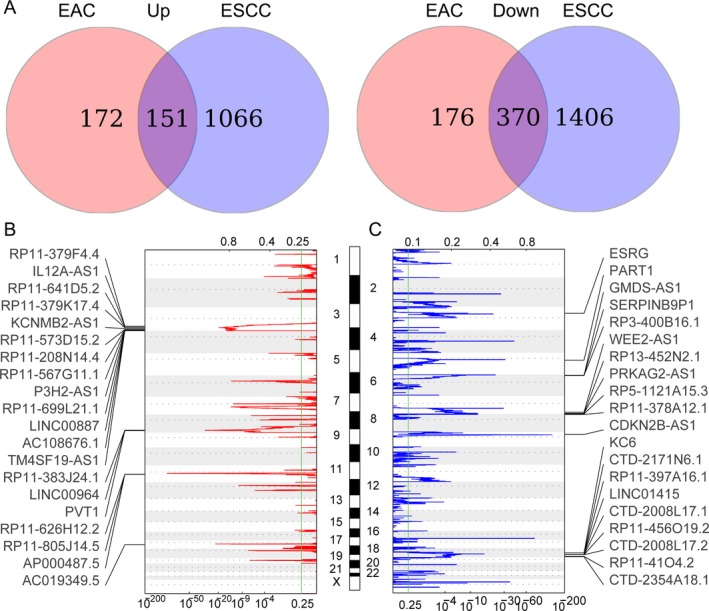
The copy number variations (CNV) of lncRNAs genomic loci in esophageal cancer. A, Venn diagram of dysregulated lncRNAs profiling in esophageal adenocarcinoma (EAC) and esophageal squamous cell carcinoma (ESCC). B, Frequency of lncRNAs genome loci copy number gain (red) in esophageal cancer tissues (top20). Each row represents an lncRNA locus, and all the rows are arranged according to the lncRNAs genomic locations. C, The frequency of lncRNAs genome loci copy number loss (blue) in esophageal cancer tissues (top20). Each row represents an lncRNA locus, and all the rows are arranged according to the lncRNAs genomic locations

Esophageal adenocarcinoma (EAC) and esophageal squamous cell carcinoma (ESCC) are two major subtypes of esophageal cancer, and they exhibit distinct molecular and genomic characterization. To stratify the lncRNAs profile in these two types of esophageal cancer, we analyzed the differentially expressed lncRNAs in EAC and ESCC using the TCGA data. And, further Venn analyses revealed that 151 lncRNAs were upregulated in both EAC and ESCC tissues, while 370 lncRNAs were downregulated in both EAC and ESCC tissues (Figure 2A). This finding indicates that the EAC and ESCC might have different lncRNAs expression pattern.

### 3.2 Copy number variations of lncRNAs loci in esophageal cancer

To date, a growing number of studies reveal that genetic alterations, epigenetic modifications, and even transcription factors are involved in regulation of lncRNAs expression in diverse cancer cells. To determine whether the genomic alterations contribute to lncRNAs alterations in esophageal cancer, we obtained the somatic copy number alterations data from TCGA. Next, each of these differentially expressed lncRNAs genomic loci copy number frequencies were calculated, and alterations in all esophageal cancer samples with *q* value <0.25 was considered as significant. The results revealed that many lncRNAs dysregulation are accompanied with CNV, such as PVT1, LINC00887 and LINC00964 with frequency gain, LINC01415 and WEE2‐AS1 with frequency loss in esophageal cancer (Figure 2A,B, and Table S2). These results indicate that genomic copy number variations are related to part of those lncRNAs dysregulation in human esophageal cancer.

### 3.3 Identifying of esophageal cancer survival associated lncRNAs

**Figure 3:**
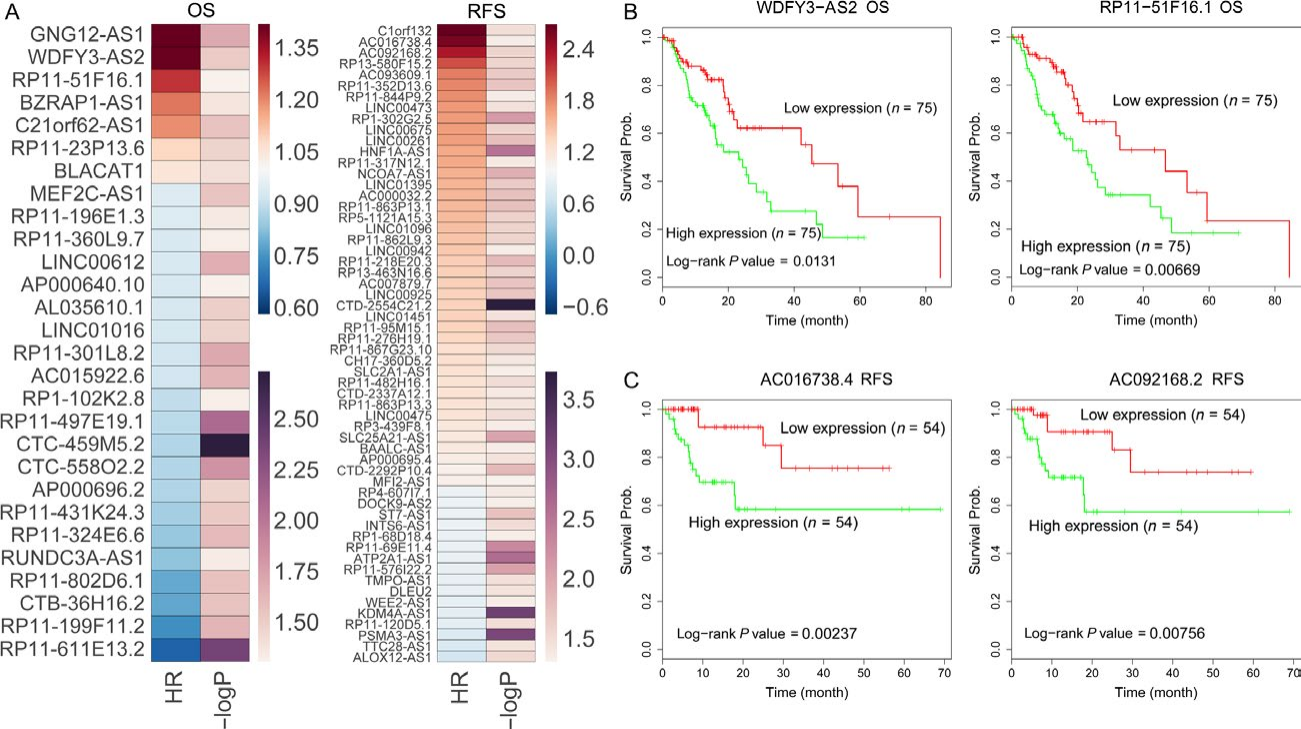
OS and RFS associated lncRNAs in esophageal cancer. A, Heat maps were drawn to show the log‐rank *P* and hazard ratio values of OS and RFS associated lncRNAs in esophageal cancer using TCGA clinical data. B, The Kaplan‐Meier curves show the OS of esophageal cancer patients with higher or lower WDFY3‐AS2, and RP11‐51F16.1PVT1 in the TCGA data. C, The Kaplan‐Meier curves for esophageal cancer patients RFS in higher or lower AC016738.4, and AC092168.2 groups in the TCGA data were examined by two‐sided log‐rank test

Recent studies have revealed that a great number of lncRNAs expression levels are associated with cancer patients prognosis, and could be useful predictors for patients overall and recurrence‐free survival time. To identify survival associated lncRNAs in esophageal cancer, we conducted univariable Cox regression analyses. The results showed that seven upregulated lncRNAs and 21 downregulated lncRNAs are significantly associated with esophageal cancer patients poorer overall survival time (log‐rank *P* < .05), and 42 upregulated and 16 downregulated lncRNAs are significantly associated with esophageal cancer patients poorer recurrence‐free survival time (log‐rank *P* < .05) (Figure 3A, Table S3). Taken WDFY3‐AS2, RP11‐51F16.1, AC016738.4, and AC092168.2, for example, esophageal cancer patients with higher WDFY3‐AS2 and RP11‐51F16.1 expression levels had shorter OS time, while esophageal cancer patients with higher AC016738.4 and AC092168.2 expression levels had shorter RFS time (Figure 3B,C). These findings suggest that these esophageal cancer survival associated lncRNAs may be useful candidates for esophageal cancer patients survival prediction.

### 3.4 DUXAP8 and LINC00460 expression are upregulated in esophageal cancer

**Figure 4:**
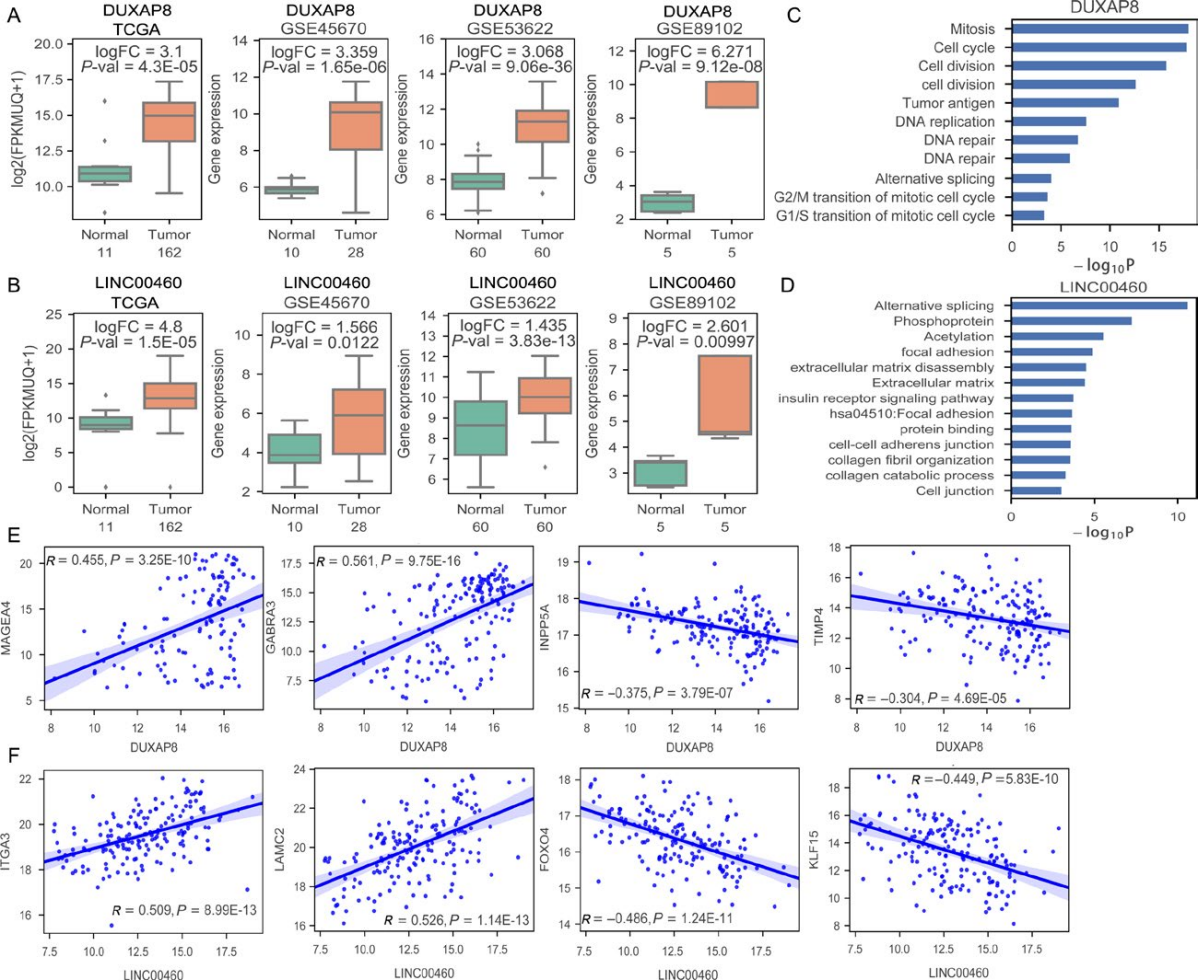
Upregulated DUXAP8 and LINC00460 associated PCGs in esophageal cancer. A and B, The fold‐changes of lncRNA DUXAP8 and LINC00460 in esophageal cancer tissues compared with normal tissues in TCGA, GSE45670, GSE53622, and GSE89102 datasets. C and D, The DUXAP8 and LINC00460 co‐expressed protein‐coding genes enriched pathways in esophageal cancer. E, DUXAP8 expression is positively related to MAGEA4 and GABRA3 expression, and negatively related to INPP5A and TIMP4 expression in esophageal cancer. F, LINC00460 expression is positively related to ITGA3 and LAMC2 expression, and negatively related to FOXO4 and KLF15 expression in esophageal cancer

To validate the analyses results, we focused on the upregulated lncRNAs that are more suitable as diagnostic molecular markers. Among these increased lncRNAs, we found that DUXAP8 and LINC00460 are consistently upregulated in TCGA data and three GEO datasets (Figure 4A,B). Interestingly, DUXAP8 has also been reported to be overexpressed in human non small cell lung cancer,^24^ gastric cancer^25^ and renal cell carcinoma,^26^ while LINC00460 expression is upregulated in nasopharyngeal carcinoma^27^ and promotes carcinogenesis in ESCC.^28^ Therefore, we chose these two lncRNAs for further analyses and validation. Firstly, we analyzed DUXAP8 and LINC00460 coexpressed protein‐coding genes in esophageal cancer and submitted DUXAP8 or LINC00460 coexpressed genes to DAVID Functional Annotation Tool for GO enrichment analysis to demonstrate their potential function and affected pathways.^29^ The results of GO and pathway analyses showed that DUXAP8 coexpressed PCGs are enriched in cell division, cell cycle and DNA repair, while LINC00460 coexpressed PCGs are enriched in alternative splicing, focal adhesion, and cell‐cell junction et al (Figure 4C,D). Interestingly, these pathways are closely associated with tumor cells proliferation and invasion, suggesting that DUXAP8 and LINC00460 might play important roles in esophageal cancer tumorigenesis and progression. Furthermore, DUXAP8 is positively related to MAGEA4, GABRA3 expression, and negatively related to INPP5A, TIMP4 expression in esophageal cancer; LINC00460 is positively related to ITGA3, LAMC2 expression, and negatively related to FOXO4, KLF15 expression in esophageal cancer (Figure 4E,F).

### 3.5 Knockdown of DUXAP8 inhibits esophageal cancer cells proliferation and invasion

**Figure 5:**
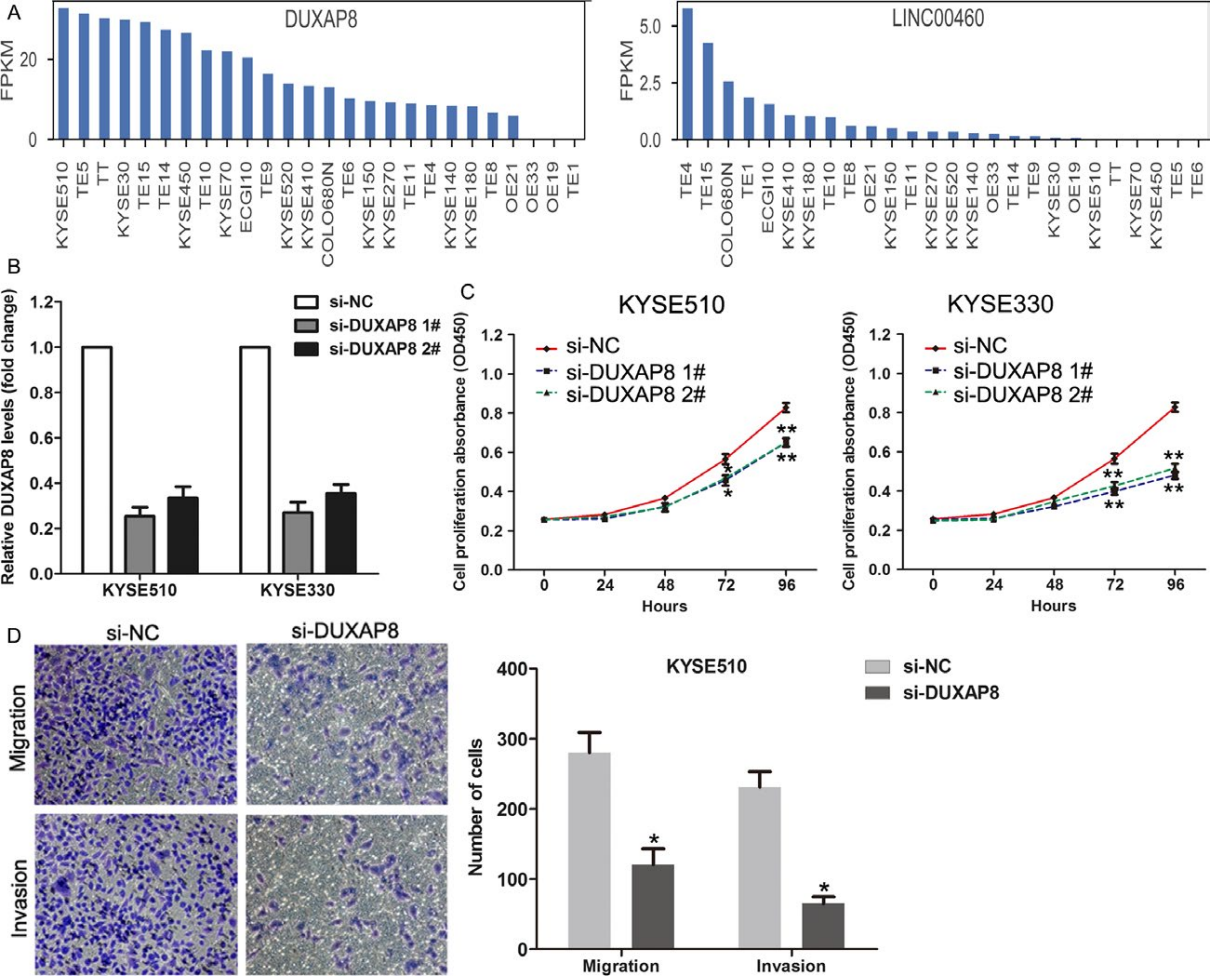
Knockdown of DUXAP8 inhibited esophageal cancer cell proliferation and invasion. A, The relative expression levels of DUXAP8 and LINC00460 in 26 esophageal cancer cell lines were analyzed using the CCLE RNA sequencing data. B, The relative expression levels of DUXAP8 in KYSE510 and KYSE330 cells after transfection with DUXAP8 siRNAs or negative siRNAs were examined using qRT‐PCR. C, The growth curves of DUXAP8 siRNAs or negative siRNAs transfected KYSE510 and KYSE330 cells were analyzed using CCK8 assay. D, Transwell assays were performed to evaluate the migration and invasive ability of DUXAP8 siRNAs or negative siRNAs transfected KYSE510 cells. **P < .01; *P < .05

To determine whether DUXAP8 or LINC00460 indeed has function in esophageal cancer, we firstly analyzed their expression levels in 26 esophageal cancer cell lines using CCLE RNA sequencing data.^30^ The results showed that DUXAP8 had relative higher expression levels in most esophageal cancer cell lines, while LINC00460 had low expression levels in most esophageal cancer cell lines (Figure 5A,B). According to these results, DUXAP8 was chosen for further function validation. Then, we transfected DUXAP8 specific siRNAs into KYSE510 and KYSE330 that had high DUXAP8 expression levels to knockdown DUXAP8 expression. The results of qRT‐PCR showed that both two siRNAs could significantly decrease DUXAP8 expression in KYSE510 and KYSE330 cells (Figure 5C). Furthermore, CCK8 assays showed that knockdown of DUXAP8 expression could impair KYSE510 and KYSE330 cells proliferation (Figure 5D) and transwell assays showed that downregulation of DUXAP8 inhibited KYSE510 cells migration and invasion ability, which is consistent with our go analyses results. These findings indicate that DUXAP8 may be important regulator in esophageal cancer.

## 4 DISCUSSION

Over the past decades, annotation of the noncoding elements in human genome reveals that tens of thousands lncRNAs are transcribed, and more lncRNAs expression is mysregulated in diverse human cancers. For example, Chen and colleagues performed a genome‐wide analysis of lncRNAs profiling and identified thousands of lncRNAs are differentially expressed in colorectal cancer.^31^ Moreover, a growing number of studies have demonstrated the function and underlying mechanisms of these dysregulated lncRNAs in cancer cells. Lu et al^32^ reported that MYC stimulates the transcription of DANCR, and inhibition of DANCR impairs cell proliferation which could be partially rescued by p21 silencing in human ovarian cancer. In addition, lncRNA MALAT1 is upregulated in hepatocellular carcinoma and exerts oncogenic function through activating Wnt pathway by induction of splicing factor SRSF1.^33^ Besides, antisense lncRNA EZR‐AS1 promotes the mobility and invasiveness of ESCC cells through enhancing SMYD3‐dependent H3K4 methylation and transcription of the EZR gene.^34^ These findings indicate that lncRNAs are novel critical regulators of tumorigenesis, and an ongoing effort is needed to identify novel esophageal cancer associated lncRNAs and determine their functional roles in esophageal cancer development.

Recently, the advances on next‐generation sequencing and microarray technique provide a chance to better understand the carcinogenesis and tumor progression at molecular level. In this study, we analyzed lncRNAs expression profiles in large cohort of human esophageal cancer specimens using RNA‐seq data from TCGA and four independent microarray datasets from GEO to identify lncRNAs related to esophageal cancer pathogenesis and survival. We found that thousands of lncRNAs are differentially expressed in esophageal cancer tissues when compared with corresponding nontumor tissues. Further somatic copy number variation analyses revealed that lncRNAs genome loci copy number amplifications or deletions are involved in part of lncRNAs dysregulation in esophageal cancer tissues, such as PVT1, LINC00887, LINC00964, LINC01415, and WEE2‐AS1. Importantly, univariable Cox regression analyses determine that more lncRNAs expression levels are significantly associated with esophageal cancer patients OS (such as WDFY3‐AS2 and RP11‐51F16.1) or RFS (such as AC016738.4 and AC092168.2). These findings in our study suggest that lncRNAs may be useful biomarker for esophageal cancer diagnosis and valuable prediction factors for esophageal cancer patients survival.

Generally, lncRNAs contribute to tumorigenesis by affecting related protein‐coding genes expression. In this study, we analyzed two upregulated lncRNAs DUXAP8 and LINC00460 co‐expressed PCGs in esophageal cancer, and found that DUXAP8 associated PCGs are enriched in cell division and cell cycle, while LINC00460 associated PCGs are enriched in focal adhesion and cell‐cell junction processes. Interestingly, these processes are closely related to cancer cells proliferation and invasion. As well as our findings, Sun et al^24^ reported that DUXAP8 promotes cell proliferation and invasion through repressing EGR1 and RHOB expression in human non small cell lung cancer; Ma et al^25^ found that DUXAP8 promotes cell proliferation through epigenetically silencing PLEKHO1 expression in gastric cancer. In addition, Kong and colleagues^27^ revealed that LINC00460 facilitates tumorigenesis by sponging miR‐149‐5p to up‐regulate IL6 in nasopharyngeal carcinoma; Liang et al^28^ reported that LINC00460 expression is upregulated in ESCC tissues and positively correlated with lymph node metastasis. Similarly, we also found that knockdown of DUXAP8 could impair esophageal cancer cells proliferation and invasion, and DUXAP8 expression is positively related to oncogenes MAGEA4 and GABRA3 expression, but negatively related to tumor suppressors INPP5A and TIMP4. These findings suggest that DUXAP8 and LINC00460 might play important roles in esophageal cancer development and progression.

In summary, our findings reveal that thousands of lncRNAs were differentially expressed in human esophageal cancer tissues compared with parental nontumor tissues. Among these dysregulated lncRNAs, many lncRNAs expression levels are significantly related to esophageal cancer patients survival time, which could be useful predictor for patients prognosis. The present study highlights the critical roles of lncRNAs in esophageal cancer, and provides a useful list of lncRNA candidates for esophageal cancer diagnostic and therapy. However, our study also has few limitations, such as, only one lncRNA candidate function was validated and its underlying mechanism is unclear, which is needed to be further investigated in our future work.

## CONFLICT OF INTEREST

No potential conflict of interests were disclosed.
